# Supplementary material for: Are carbon emissions trading and green financial instruments synergistic? -Comprehensive quantitative research based on content analysis
Source: PLoS One. 2024 Mar 7;19(3):e0298601. doi: 10.1371/journal.pone.0298601 (PMC10919601; doi:10.1371/journal.pone.0298601)
Supplement: S1 Data — (ZIP) [file pone.0298601.s001.zip › Data/地级市Z_F工作报告.zip!/╕ú╜¿/╚¬╓▌╩╨/└┤╫╘╣1⁄2╓┌║┼í╛┬φ┐╦╩2╛▌═°í┐_2018.docx]

　各位代表：

　　现在，我代表市人民政府向大会报告政府工作，请予审议，并请政协委员和其他列席人员提出意见。

　　一、2017年政府工作回顾

　　一年来，在中共泉州市委领导下，市人民政府认真学习贯彻党的十九大精神，学习贯彻习近平新时代中国特色社会主义思想，全面落实中央、省和市委决策部署，着力稳增长、控风险、谋长远，经济运行稳步回升，金融风险有效化解，产业格局更加优化，实现地区生产总值7533亿元、增长8.3%（预计，下同），主要经济指标保持较快增长；集中力量办成一批影响全局、关系民生的大事要事实事，有力地推动泉州发展迈上新台阶。

　　——“三大板块”渐成格局，实体根基更加坚实。全市上下大抓发展、大抓龙头、大抓高科技产业，晋华集成电路、中化乙烯、三安高端半导体等投资数百亿元的重特大项目强力推进，传统、重化、高新产业在手大项目投资总额分别达521亿元、730亿元和1089亿元，集成电路、半导体等产业呈现大好发展态势，“三大板块”实现从构想到路线图，到落地见效、初步成形。

　　——古城新城齐头并进，城市颜值更加靓丽。开展国家生态修复、城市修补试点，在“润物细无声”的微改造中，古大厝修旧如旧，古街巷活化保护，泉州古城逐步成为见人见物见生活的“活样本”。海丝新城加快环湾向湾生长，人气、业态、服务加速集聚；绿化景观明显提升，精心打造山线水线绿道，织就美丽风景线，串起城市“慢生活”。环湾建成区达220平方公里。

　　——文脉传承深入人心，文化自信更加坚定。以“古泉州（刺桐）史迹”申遗为契机，大力推进历史文化遗产保护、挖掘和整理；守护好古城、古镇、古村落，越来越成为全市上下共同的文化使命和行动自觉。成功举办金砖国家治国理政研讨会、第三届海丝国际艺术节。晋江成功申办第18届世界中学生运动会。永春苦寨坑原始青瓷窑址当选年度中国十大考古新发现。

　　——惠民举措叠加推出，人民生活更加幸福。坚持把七成以上的地方财力用于民生，落实市委“四心”工程，实施教育、医疗、养老、基础设施等补短板项目132个。启动泉州“XIN”行动，聚焦安置房办证、中心城区防洪排涝等10个问题，从体制机制入手，突破长期想解决而没有解决的难题。扎实推进精准扶贫、精准脱贫，现有的建档立卡贫困村和贫困人口实现按时退出。加快建设国家生态文明试验区，空气、水质量保持优良水平。荣膺国家森林城市、全国未成年人思想道德建设工作先进城市，蝉联全国文明城市、国家卫生城市、综治最高奖“长安杯”。

　　一年来，我们主要抓了以下工作。

　　（一）把稳定经济增长作为重中之重。政企联手、巩固工业、大抓三产，规模以上工业、第三产业增加值分别增长8.2%和10.5%。全力稳企业发展。落实市委“促进项目落地 帮扶企业发展”活动周，出台稳定工业、奖励用电、扶持外贸等专项政策，减轻企业负担42.6亿元。举办全国性特色商品博览会7场，开展泉州品牌“中国行”“境外行”展销活动52场。全力稳项目投资。落实市委“项目攻坚年”活动，市领导分片区一线推进，重大项目实行指挥部体制，396个在建重点项目完成投资1271亿元，中化乙烯、通达三期等92个项目开工建设，西人马高端芯片、晶安光电三期等73个项目建成或部分建成，晋华集成电路主厂房封顶。建立重大产业项目招商协调机制，新引进项目323个、总投资2031亿元。全市完成固定资产投资4160亿元，其中工业投资占39.5%。全力稳金融运行。认真落实金融风险防控属地责任，创新困难企业帮扶措施，坚决打击、联合惩戒恶意逃废债，不良贷款率控制在较低水平。

　　（二）把提高供给质量作为主攻方向。实施产业转型升级路线图，力促产业向中高端迈进。深化国家自主创新示范区建设。完善“1+X”政策体系，首创31项新举措。坚持一手抓高新技术产业发展，一手抓传统产业改造提升，新认定高新技术企业169家、科技小巨人领军企业198家。整合晋江集成电路、南安半导体、安溪湖头光电等3个园区，获批设立省级半导体高新技术产业园。丰泽入选国家双创示范基地。实施市委“人才港湾”计划，认定高层次人才2415人，新增院士工作站12家；设立“一站式”人才服务平台，建成人力资源服务产业园。实施“中国制造2025”城市试点示范。完成“百千5311”工程，培育“数控一代”示范产品138个，列入国家级智能制造项目5个、服务型制造示范项目3个、工业设计中心2家。实施重点技改项目238个，全市完成技改投资990亿元。加强商标品牌建设，获评“中国商标金奖”企业1家、国家质量标杆企业1家，制（修）订标准132项。新增中国专利奖优秀奖4项。开展“第三产业提升年”活动。培育认定百强企业。新增3A以上物流企业15家，全市快递业务量7.3亿件，占全省近一半；国际邮件互换局获批设立，石狮国际快件监管中心投入运行。全市电商交易额4000亿元、增长42%，网商（虚拟）产业园集群注册发照1万多家，泉智汇、泛家居等平台投入试运行。全年接待游客5460万人次、旅游总收入840亿元，分别增长21%和28%，列入全国优选旅游项目5个。启动“泉州老字号振兴计划”。“接二连三”发展特色现代农业。完成农林牧渔业产值355亿元。农业“一区两园”完成投资15.3亿元，安溪获批创建国家级现代农业产业园。新增农业新型经营主体500多家、“三品一标”认证38个。扶持农产品生产加工龙头企业30家，培育农业物联网示范企业12家。基本完成农村土地承包经营权确权登记颁证工作。全市远洋渔船57艘，捕捞产量8万吨。

　　（三）把全面深化改革作为永续动力。发挥“金改区”“综改区”等国家级改革试点效应，在重点领域实现多点突破。提升金融服务实体经济水平。创新供应链融资模式，小微企业贷款2100亿元、增长10.7%。“无间贷”模式成为全国典型。新增境内外上市企业3家、“新三板”挂牌企业8家；海峡股权泉州交易中心设立“青创板”，累计挂牌企业606家。激发民间投资活力。新兴产业、集成电路等基金为企业提供融资59亿元。建立PPP项目审批绿色通道，落地项目26个、总投资316亿元，其中纳入财政部示范项目8个。深化工商登记制度改革。率先启动“多证合一”，实行工商简易注销，“双随机”抽查实现全覆盖，工商注册便利化工作获国务院通报表扬。市场主体总量达68万家，位居全省首位。完善生态文明体制机制。深入推进晋江自然资源资产统一确权登记、永春空间规划编制等省级试点；完成林权收储、环境治理市场主体培育等8项改革任务。泉州开发区被联合国工发组织授予“绿色开发区”。实施国有企业改革重组。整合40家国有企业，组建城建、文旅、金控、交通发展、水务5家市级国资集团。深化海丝先行区开放合作。全市完成进出口总额1569亿元，与海丝沿线国家和地区贸易额720亿元。与马来西亚古晋南市建立友好城市关系。泉州晋江国际机场旅客吞吐量523万人次、增长38%。完成港口吞吐量1.3亿吨、集装箱217.2万标箱。泉金航线运送旅客13.8万人次。石井口岸正式对外开放。

　　（四）把开展生态修复、城市修补作为重要抓手。完成环泉州湾城乡一体化规划，全面推行城市设计。突出古城保护提升。启动“七个一”示范工程，修缮一批古大厝、特色建筑，“东亚之窗”、美食城等项目进展顺利。西街东段、中山中路实施分时段交通限行和精细化管理。开展“社区营造”试点。完善城市交通体系。兴泉铁路、福厦客专开工建设，泉厦漳城市联盟路、城东至北峰快速通道、动车站综合交通枢纽等项目加快推进，厦沙高速泉州段建成通车。市区建设公共停车设施33个，新增停车泊位5000多个。推行公交一元票价制。市区公共自行车“小黄人”累计建设站点470多个，骑行总次数3200多万人次。“点线面”推进绿化提升。精心打造高速出入口、动车站等一批关键节点绿化工程。改建特色树种景观路14条；山线绿道示范段，以及沉洲、汀洲、白水营等水线绿道公园建成开放。全市完成绿化改扩建面积65万平方米。结合“创城”“创卫”开展环境综合整治。认真执行市人大《关于加快全市垃圾分类处理利用的决议》，启动生活垃圾强制分类试点，大件垃圾处理站投入运行。疏堵并举治理占道经营等城市乱象，环境卫生考评实现城乡全覆盖。标准化改造中心市区农贸市场7个。拆除“两违”557.5万平方米。整治市区内涝积水点54处。推进城市执法体制改革，整合175项集中行政处罚权职能。

　　（五）把保障改善民生作为第一追求。完成33件市委市政府为民办实事项目。打好脱贫攻坚战。认真落实“六个精准”要求，扶持30个市级扶贫重点乡镇、300个扶贫重点村发展,实现贫困线和低保线“两线合一”。创新帮扶机制，形成社会扶贫“三百”工程、安溪乡镇扶贫开发协会全覆盖、南安光伏扶贫等典型做法。农村危房改造980户，造福工程搬迁1.1万人。办好人民满意教育。实施“领航团队”培养工程。新增公办幼儿园学位1万个、中小学学位1万个。深化职业教育改革，启动技能名师工作室建设，壮大“双师型”教师队伍，现代学徒制试点基本覆盖主要产业，建成福建省大学生创新创业基地（泉州）。抓好关心下一代工作，发挥好青少年社会教育活动中心的校外阵地作用。加快卫生计生事业发展。深化公立医院改革，推行目标管理责任制和院长年薪制，启动“名医带徒”工作。实施基层医疗卫生服务能力三年提升工程。全市新增医疗机构床位2512张。建立统一的城乡居民基本医保制度。加大妇幼医疗资源配置，服务“全面两孩”政策。完善养老服务体系。新建农村居家养老服务站135个，建设提升农村老年体育活动中心30个，市社会福利中心开工。全市新增养老床位1460张，享受政府购买信息化养老服务的人数超3万。加强老龄、老干部工作。推进文化繁荣发展。积极践行社会主义核心价值观。认真落实《泉州市海上丝绸之路史迹保护条例》，全力推进“古泉州（刺桐）史迹”申遗，高标准实施16个申遗点文物本体修缮和周边环境整治，完成国际专家现场考评阶段性任务。公共文化中心主体工程完工，市少儿图书馆正式开放。建成24小时街区自助图书馆46个、村（社区）文化服务中心示范点24个。成功举办联合国教科文组织青年创意与遗产研习班、“中国白”国际陶瓷艺术大奖赛、环泉州湾国际公路自行车赛。第十三届全运会收获金牌8枚，金牌和奖牌数均居全省首位。加强社会保障工作。城镇新增就业12.6万人。城乡医疗、养老保险待遇水平稳步提升，全民参保登记计划全面实施。建立特困人员救助供养制度。及时出台房地产调控政策，实行限房价竞地价、备案价格会商、公证摇号销售等措施，严控投机炒房行为，稳定房地产市场。基本建成保障性安居工程2.6万套，改造石结构房屋3600万平方米。德化进城务工人员安居项目荣获中国人居环境范例奖。深化生态环境整治。以中央、省环保督察为契机，狠抓150项任务整改，开展环保设施与能力建设大会战，建立生态环境保护工作职责体系。认真执行市人大《关于进一步加强全市流域水环境保护的决议》，全面推行河长制，所有集中式饮用水源地和主要干流水质达标率保持100%。启动农村污水垃圾治理三年行动，266个行政村新建污水处理设施，建设改造三格化粪池6.5万户，新增480个行政村实现垃圾常态化治理，持续开展生猪养殖和农业面源污染整治。完成植树造林12万亩、水土流失治理31万亩、矿山生态修复313万平方米。永春流域综合治理荣获中国人居环境范例奖，成为全国典型案例。创新社会治理体制。推进“雪亮工程”，坚决打击和防范违法犯罪活动，完善矛盾纠纷多元化解机制，提升平安泉州建设水平。扎实开展食品药品安全综合监管，加强食用农产品入市质量安全检测，深入推进“餐桌污染”治理。开展安全生产大检查，深化隐患排查治理体系建设，安全生产形势持续稳定好转。完善国防动员机制，把支持深化国防和军队改革当作分内的事，助推驻泉部队现代化建设，实现双拥模范城“满堂红”。民族宗教、统计、物价、粮食、方志、档案、人防、支前、气象、地震以及工会、青少年、妇女儿童、残疾人、红十字会等工作都取得新成效。

　　（六）把增强政府履职能力摆上突出位置。严格落实中央八项规定及实施细则精神。深入推进“两学一做”学习教育常态化制度化。自觉接受市人大及其常委会监督，依法执行市人大及其常委会决定决议，主动配合市人大依法履职，办理人大代表建议383件，满意和基本满意率达100%。支持市政协履行职能，主动接受民主监督，把协商民主贯穿政府履职全过程，办理政协提案596件，满意和基本满意率达100%。坚持依法治市，深化府院联席会议机制，法治政府、法治社会建设互相促进。全面推行权责清单制度，进一步规范行政审批自由裁量权，实施“一趟不用跑”“最多跑一趟”改革，推行周末收件、24小时自助服务等便民措施，深化国家级电子证照试点，行政审批制度改革获国务院通报表扬。加大效能督查力度，加强廉政、监察、审计等工作。

　　各位代表，过去一年成绩来之不易，这是省委、省政府和市委正确领导的结果，是全市人民齐心协力、团结奋斗的结果。在此，我谨代表市人民政府，向全市广大干部群众，人大代表、政协委员，各民主党派、工商联、无党派人士、各人民团体、离退休老同志，向驻泉省部属单位、驻泉部队、武警官兵，企业界人士和来泉创业务工人员，向广大港澳同胞、台湾同胞、海外侨胞、海内外朋友们，表示衷心的感谢和崇高的敬意！

　　同时，我们清醒地认识到，工作中还存在许多不足和困难。主要是：发展不平衡不充分问题仍然突出，传统产业转型升级任重道远，高新技术产业规模不大，第三产业比重偏低、层次不高，科技创新、人力资源、职业教育支撑不足。城市规划建设管理水平有待提升，基础设施和公共配套相对滞后。统筹城乡力度有待加大，民生保障存在不少短板。资源环境约束趋紧，生态保护、污染防治、节能减排工作仍需持续用力。政府职能转变不够到位，一些工作人员的素质、能力和作风不适应新要求，营商环境仍需优化。对此，我们要高度重视，下更大力气加以解决。

　　二、2018年工作目标任务

　　党的十九大是我们党和国家发展史上一个重大里程碑，正式宣告中国特色社会主义进入新时代，作出了社会主要矛盾转化的重大论断，鲜明提出了习近平新时代中国特色社会主义思想，指明了实现“两个一百年”奋斗目标和中华民族伟大复兴中国梦的前进方向。做好2018年政府工作：必须全面贯彻党的十九大精神，以习近平新时代中国特色社会主义思想为指导，坚持稳中求进工作总基调，坚持新发展理念，按照高质量发展的要求，主动融入新福建建设大局，突出转型攻坚、聚力跨越主线，深入实施“十三五”规划，深化供给侧结构性改革，全力推进产业升级、城市建设、改革开放、民生补短，决胜全面建成小康社会，加快打造“创新、智造、海丝、美丽、幸福”的现代化泉州。

　　经济社会发展主要预期目标是：全市生产总值增长8.7%以上，农业总产值增长2.5%左右，工业增加值增长7.8%左右，第三产业增加值增长10.4%左右；一般公共预算总收入增长5.5%左右，一般公共预算收入增长6.5%左右；固定资产投资增长13%左右；实际利用外资增长2%左右，出口增长2%左右；社会消费品零售总额增长11%左右，居民消费价格涨幅控制在3%左右；居民人均可支配收入增长7%左右；完成节能减排任务。

　　为此，我们要在市委统一领导下，把高质量发展与实现赶超有机统一起来，推动经济社会更好更快发展。在“稳”的基础上着力于“进”，强化工业支撑，扩大三产贡献，加快经济提速扩量；在转型升级上迈开大步，推动“三大板块”成形成势，致力构建“1234”现代产业体系；在城市建设上加速提档，全面推开环湾区域片区更新、景观塑造、修复修补；在民生领域攻坚补短，深入推进“四心”工程，滚动实施“XIN”行动，抓好防范化解重大风险、精准脱贫、污染防治三大攻坚战，努力满足人民群众美好生活需要。

　　（一）突出实体为本，着力建设现代化经济体系

　　始终把发展着力点放在实体经济上，从项目支撑、技术支撑、人才支撑入手，推动“三大板块”竞相发展，全力打造高端、高质、高新的制造强市。

　　巩固扩大传统产业领先优势。一是坚持用新技术、新模式、新业态改造提升传统产业。以智能化改造、供应链整合、新材料应用等为主攻方向，抓好安踏一体化、九牧智慧工厂、百宏差别化纤维等项目。建立适用传统产业转型升级的技术需求库、企业后备库和高端转化平台。二是加快完善产业生态圈。鼓励龙头企业，通过技术升级、主业扩张、兼并重组等方式发展壮大，建设一批核心工厂、高端工厂，打造一批具有国际影响力的大企业集团。鼓励中小企业向“专精特新”方向提升，培育一批单项冠军企业和产品。三是争创全国质量强市示范城市。大力弘扬工匠精神，开展质量品牌提升行动，树立“泉州制造”品牌形象。

　　延伸拓展重化产业链条。统筹泉港、泉惠石化园区发展，发挥联合石化、中化两大龙头的带动效应，吸引更多企业进入石化深加工领域，大力发展石化新材料和精细化工。重点抓好中化乙烯、天佑能源、中仑塑业、天骄化学等项目，启动中化专属园区建设。加快现有装备产业整合提升，坚持以市场引技术、引项目，推进国家级特种机器人检测评定中心、嘉泰数控（二期）、明匠智能制造等项目建设，加快新能源汽车推广应用和产业发展，建设南安、晋江、洛江、安溪等智能装备产业园。

　　培育壮大高新技术产业。紧盯市场前景和科技前沿，坚持引龙头、聚项目，着力打造若干个全国重要的新兴产业基地。一是培育集成电路产业。力争晋华集成电路一期投产，加快矽品、芝奇等项目建设，构建“设计—制造—封装测试—装备材料—终端应用”全产业链。二是培育化合物半导体产业。抓好三安高端半导体等项目，对接引进更多配套企业，加强与国家大基金合作建设泉州“芯谷”。三是培育石墨烯产业。立足泉州产业发展需求，推动发展石墨烯材料提炼和石墨烯纤维材料、高透气防水杀菌材料、防腐涂料等，加快产业化应用步伐。

　　创新发展现代服务业。深化“第三产业提升年”。一是稳步推进主辅分离，加快发展研发设计、信息服务、供应链管理等服务外包，培育20家服务型制造示范企业。二是深化“生产基地+物流+互联网”。支持龙头企业建设智能仓配一体化园区。加快安通综合物流园、中农批国际物流港、南方水产城等项目建设，建好陆地港、天地汇等一批第三方、第四方物流，深入开展“无车承运人”试点，有效引导外挂车船回流。发展壮大垂直供应链电商平台，培育东海跨境电商服务生态圈。推动石狮服装城争创市场采购贸易方式试点单位。三是紧抓文化旅游业这一爆发点。围绕打造“世界海丝文化休闲旅游目的地”，按照“亮点在古城、厚度在山海、空间在生态连绵带”的全域旅游布局，实施“六大旅游产品”培育计划，加快海丝古港旅游综合体策划，用心塑造“古泉州（刺桐）史迹”旅游品牌；抓好八仙过海、小岞生活艺术岛、海丝茶源、岵山古镇、石牛山等项目，高起点构建全市智慧旅游平台。四是推动文体娱乐、健康养老、家政护理等生活性服务业，向精细和高品质转变，促进消费结构升级。

　　培育发展新动能。一是争创“中国制造2025”国家级示范区。深化智能制造示范创建工程，实施重点技改项目200个，建设数字化示范车间和智能工厂15个以上，推广智能装备和工业机器人1000台（套）以上。加快发展数字经济，推动互联网、大数据、人工智能同实体经济深度融合。二是推进国家自主创新示范区建设。制定泉州片区发展规划，提升高新区发展质量。鼓励企业加大研发投入，强化知识产权创造、运用和保护，组织实施重大科技专项20个，新增高新技术企业120家、科技小巨人领军企业50家以上。高度重视军民融合产业发展，鼓励更多企业参与军品研发生产。三是进一步发挥政企互动好传统。旗帜鲜明支持民营企业发展，激发和保护企业家精神，深化企业家素质提升培训计划，加强对新一代企业家的培养，做好传帮带工作，打造一支有义、有根、有智、有梦的企业家队伍。四是深入实施市委“人才港湾”计划。完善育才引才聚才政策体系，加强人才社区、双语学校、国际医疗等高端配套，解决人才后顾之忧，努力建设人才的梦想港湾、事业港湾、生活港湾。

　　发挥投资的关键性作用。持续开展“项目攻坚年”活动，按照“五个一批”要求滚动推进，实施在建重点项目423个、总投资8558亿元，力争开工项目70个、竣工70个，全年完成重点项目投资1400亿元以上。注重投资质量，加强重大项目前期论证，实施大招商、招大商“一把手”工程，强化专业招商队伍建设，完善招商考核机制，力促重大产业项目接续落地。

　　（二）突出乡村振兴，加快推进农业农村现代化

　　实施产业振兴工程。一是落实粮食安全行政首长责任制。粮食总产量稳定在72万吨以上，基本建成52万吨标准化仓容。二是落实农业功能区制度。围绕茶叶、水果、食用菌等特色产业发展，创建省级以上现代农业产业园8个，建设安溪、永春2个省级智慧农业园。新建农业标准化基地300个，新增“三品一标”认证20个。深入实施农产品质量安全行动计划，加强可追溯平台建设。大力发展林下经济。三是推进一二三产业融合发展。扶持一批农业产业化龙头企业、农业物联网示范企业和乡村旅游示范点，发展田园综合体。培育壮大远洋渔业、水产品精深加工、海洋生物医药产业，建设渔港经济区。

　　实施宜居环境工程。一是深入推进美丽乡村建设。摒弃“农村脏一点不要紧”的观念，组织实施265个农村污水治理项目，推动所有行政村实现生活垃圾常态化治理。抓好22个小城镇试点，培育发展特色村镇，建设一批环境整治示范村、宜居村。二是加强农业面源污染治理。健全农业投入品减量使用制度，深入开展化肥和农药零增长行动。推行规模养猪场建档立卡管理。三是完善农村生产生活基础设施。抓好白濑水利枢纽、惠女至菱溪引调水、南安“两溪一湾”整治、永春马跳水库等重大水利项目。提级改造农村电网。建立农村公路“路长制”，加强公路安全生命防护工作，改造一批危病桥，建好、管好、护好、运营好农村公路。

　　实施农民增收工程。一是发挥新型农业经营主体的带动作用，引导更多农民抱团致富，新增农民合作社、家庭农场400家以上，扶持市级示范社和示范农场50家。二是增加农民财产性收入。推进农村土地“三权分置”，加快土地流转，促进农民增收，发展适度规模经营。深化集体林权制度改革，抓好国有农场、林场改革。三是拓宽村财收入。扶持200个经济薄弱村发展，综合实施资源开发、资产租赁、股份合作等创收举措，开展农村集体产权制度改革，抓好晋江、永春、泉港等试点，多途径壮大集体经济。

　　确保小康路上一个都不掉队。把扶贫工作锲而不舍抓下去，保持原有扶贫政策不变、支持力度不减，形成长效化扶贫机制。致力增强造血功能，支持通过就业创业、入股分红等渠道实现稳定收入，改善提升1300户建档立卡贫困户的住房条件，大力推行精准扶贫医疗叠加保险，让贫困户有尊严脱贫、稳定脱贫。更加关注贫困线边缘的低收入群体生产生活，逐步提高低保标准，提升帮扶实效，筑牢民生兜底保障安全网。

　　完善乡村治理体制。加大城乡统筹力度，建立健全城乡融合政策支撑体系。以扩大有序参与、推进信息公开、加强议事协商、强化权力监督为重点，完善村民自治制度。抓好农村移风易俗试点。突出政治功能，加强农村基层组织建设，培养一支懂农业、爱农村、爱农民的“三农”工作队伍。

　　（三）突出机制创新，全面深化改革开放

　　推进重点领域先行先试。深入实施国家级、省级各项改革试点，进一步拓展“晋江经验”内涵，努力打造具有泉州特色的改革体系。加大实体经济的金融服务力度，发挥好泉州金融服务平台、中小微企业信用信息平台作用，推广“松散型”银团贷款、无还本续贷等做法，既保障重大项目、重点企业，又支持中小企业、民生项目的融资需求；大力发展科技金融、绿色金融等新型业态；扩大企业直接融资规模；加大不良贷款处置力度，切实维护良好金融生态。深化财税体制改革，壮大地方财源，推进产业股权投资基金市场化运作，加大PPP模式的推广应用。深入开展工商登记制度改革，探索实施“证照分离”，打造市场准入便利化链条，营造大众创业、万众创新的良好氛围。推动五大国资集团实体化、商业化运营，在做开发、做实业、做民生、做金融等方面发挥更大作用，加快构建国资监管新体系，力争市属国有企业资产达1200亿元。深化价格、供销等领域改革。

　　推动形成全面开放新格局。深入实施海丝先行区建设行动方案和发展规划。坚持“引进来”和“走出去”并重，大力发展外贸综合服务企业，支持品牌企业开展国际化经营，进一步提升出口质量和附加值。举办第四届海丝国际品牌博览会。鼓励企业在“一带一路”沿线国家和地区，建设营销网络和经贸合作园区，搭建集群式“走出去”国际合作平台。发挥综合保税区等政策优势，优化口岸功能布局，抓好石湖5#、6#和石井16～19#等泊位建设，增开内外贸航班航线，争取更多货源从泉州港进出。主动复制自贸区创新政策，提高贸易便利化水平。加强与港澳侨交流合作，实施侨务“三新”工程，力争上线运行南洋华裔族群寻根谒祖综合服务平台，涵养新华侨华人、华裔新生代资源。

　　深化对台交流合作。认真贯彻中央对台大政方针，多做文化认同、民心相通的工作。发挥台商投资区等载体作用，深化泉台集成电路、精密机械、医疗健康等产业对接，大力吸引台湾人才来泉就业创业。

　　（四）突出品位品质，提高城市承载力和宜居度

　　坚持谋定而后动，以开展生态修复、城市修补为抓手，下大功夫修整古城，用大手笔建设新城，花大心思提升管理，让泉州这座城市个性更明显、气质更凸显、魅力更彰显。

　　古城要更有古早味。一是原真性保护。制定传统建筑修缮技术导则，疏解古城功能，恢复历史空间。在西街开展以危旧房屋修缮为主题的“点亮家园”活动，修缮中山路风貌建筑，挂牌保护669幢传统历史建筑，做到“原址、原状、原物、原汁原味”。二是活态化利用。盘活古城公房、古大厝资源，科学布局本地文创产业，建设旅游集散和游客服务驿站，培育精品酒店群和非遗活态展示空间，优化古城业态。重塑小山丛竹、一峰书院等原貌。办好海丝古城徒步穿越活动。三是功能性提升。按照海绵城市理念建设综合管线，启动中山路试验段，开展象峰巷、三朝巷等街巷微改造。构建“公交+慢行”交通方式，打造公交“小蓝”、社区巴士“小白”、自行车“小黄人”、三轮车“小红”等“多彩交通”。四是生态型修复。内沟河是城市水系的重要组成部分，流淌着泉州的历史，见证着古城的变迁。要认真实施《泉州市市区内沟河保护管理条例》，以八卦沟、笋浯溪、百源川池为重点，分期推进水环境整治，打造水清、岸绿、景美的城市地标线。通过见缝插绿、拆改腾退，建设一批口袋公园、袖珍绿地。

　　新城要更有现代感。一是组团式开发。加快推进东海、城东、晋东、台商区、北峰、江南等组团的片区开发和改造，完善居住、商务、休闲、文教、医疗等功能，培育新城核心区，形成“一湾两江”城市展示面。二是体系化推进。加快兴泉铁路、福厦客专、泉厦漳城市联盟路、城东至北峰快速通道、东海通道、动车站综合交通枢纽等项目进度，抓紧金屿、百崎、武荣通道等项目前期工作，完善环湾交通体系。规划建设生态连绵带，依托现有山水脉络，用绿道串起山林、水体、湿地、田园等自然资源，形成相互衔接、连绵成片的生态体系，让好山好水好风光融入城市。坚持“点线面”结合，加快交通枢纽、重要节点，以及“居民常走、客人常走”门户廊道的景观提升，建成水线绿道系统和山线绿道二期，抓好晋江、洛阳江下游两侧规划建设及流域综合整治。三是标准化配套。加快完善市政设施，建成投用公共文化中心，建设提升防洪排涝设施，进一步完善供电、供气、供水、排水系统。加强人行道、自行车道同步配套。四是统筹化布局。启动新一轮城市总体规划修编，推行“多规合一”，以城市设计为抓手，推进环湾各组团在项目规划、功能、标准、时序等方面的相互衔接，形成同城化发展合力。高标准规划建设南北翼新城，抓好泉州“芯谷”南安园区、泉港石化安全控制区等项目，打造“产、城、人”融合发展样板。支持县域大城关建设。

　　管理要更加精细化。一是从市民感受最直接的环节抓起。抓好背街小巷修补、工程文明施工、户外广告整治、共享单车停放、红绿灯配时、渣土车运营、垃圾箱分布、夜景照明等精细管理。认真落实市人大《关于加快全市垃圾分类处理利用的决议》，扩大试点范围，实现中心市区垃圾分类大分流。持续开展“无违建”创建活动，推进“两违”综合治理。二是强化城市管理保障。深化执法体制改革，实现“一支队伍管到底”。推动市政公用设施管养分离，加强市场化运作。拓展“数字城管”平台功能，推行城市智慧管理和全面监督。稳妥推进市容环境、交通秩序等政府立法工作。三是倡导共建共管共享。完善家园共造机制，扩大“社区营造”试点，落实“门前三包”制度，提升住宅小区物业管理水平。

　　（五）突出传承创新，增强泉州文化软实力

　　文化是一座城市的灵魂。要进一步传承活化千年的海丝文化、闽南文化，让泉州人身上的文化基因代代相传、薪火永续，让文化走进市民生活，走出家门国门，走向光明未来。

　　坚守核心价值体系。坚持全民行动、干部带头，坚持从家庭做起、从娃娃抓起，深化“家+文化”建设，让社会主义核心价值观内化于心、外化于行。实施公民道德建设工程。推动全国文明城市创建常态化机制化，支持发展志愿服务组织。强化守信激励和失信惩戒联动机制，争创国家社会信用体系建设示范城市。

　　保护传承传统文化。全力推动“古泉州（刺桐）史迹”申报世界文化遗产，发挥16个申遗点带动作用，健全文化遗产保护长效机制。建设中国海上丝绸之路博物馆。开展优秀传统文化传承发展示范城市创建活动，实施闽南文化保护示范工程，加强历史文化名镇名村、传统村落保护，建成泉州非物质文化遗产馆。抓好古籍文献收集整理。完成《泉州市志》第二轮修编。争创中国工艺美术之都。

　　繁荣发展先进文化。深化文化体制改革。完善提升基层公共文化设施，争创国家公共文化服务体系示范区。加强新时代文艺精品创作，实施“戏曲名家收徒传艺”计划，全面普及公益性文艺惠民演出。大力发展文化产业、文化市场，培育动漫、创意设计、文物博物等新型文化业态。深化全民健身计划，办好国际大体联足球世界杯预选赛、环泉州湾国际公路自行车赛，筹备世界中学生运动会，推动体育制造业与体育服务业协同发展。

　　（六）突出绿色发展，加快建设美丽泉州

　　健全生态文明制度。深入推进国家生态文明试验区建设，完成污染物排海总量控制、用能权有偿使用交易等15项综合试验任务，推进制造业转型升级与生态文明建设形成正向互动，创造一批在全省、全国可复制推广的成果和经验。强化生态环境保护工作职责，认真落实中央、省环保督察整改任务，严格执行红线管控措施，持续完善“两江”流域生态保护补偿机制。

　　着力解决环境突出问题。按照市人大《关于进一步加强全市流域水环境保护的决议》要求，实施一批跨境流域、重点流域、小流域及黑臭水体、海漂垃圾整治项目，确保全面消除劣V类水体。因地制宜建设安全生态水系。认真落实“河长制”“湖长制”，以流域治理为龙头，统筹山、水、林、田、湖、草等系统治理，打造各具特色、清新自然、富有野趣的流域环境生态样板。深化环保设施与能力建设攻坚，抓好水、垃圾、危废处理设施以及环境监测能力4方面136个项目。全面落实大气、土壤、重金属污染防治工作。坚持不懈推进“厕所革命”。实施森林质量提升工程。

　　推动节约循环发展。开展能效对标活动，完善重点用能企业的能源监测、审计和管理体系。建好德化陶瓷、泉港石化等国家级循环经济园区。加快淘汰落后产能。全面推进碳排放权交易市场建设。依法清理处置闲置土地，盘活城乡低效建设用地。发展装配式建筑。争创国家节水型城市。

　　（七）突出人民主体，不断增进民生福祉

　　坚持以人民为中心的发展思想，以造福人民为最大政绩，对人民群众的悲欢冷暖要感同身受，对民生实事要一件接着一件办、一年接着一年干，切实把市民的期待变成我们的行动，把市民的希望变成生活的现实。

　　努力让群众享有更好的教育。对照义务教育学校管理标准，深入实施“全面改薄”，推进优质高中建设、“小片区管理”，着力解决中小学生课外负担重、“择校热”“大班额”等突出问题。新改扩建公办幼儿园70所，全市新增学位1万个，普惠性幼儿园覆盖率达75%，解决好婴幼儿照护和儿童早期教育服务问题。提高职业教育与产业发展契合度，重点培育39个服务产业的特色专业群，创建示范性现代职业院校，实现中等职业学校达标全覆盖；推动普通本科高校向应用型转变。实施基础教育“领航团队”培养工程和职业教育名师“百人计划”，加强师风师德建设。办好继续教育、特殊教育，抓好老年教育，建设老年大学新校区。支持和规范社会力量兴办教育。加强未成年人思想道德建设和心理健康服务。做好关心下一代工作。

　　努力让群众享有更高水平的医疗服务。深化医改工作，提高公立医院综合改革实效；以医联体为突破口，组建城市医疗集团、县域医共体、专病专科联盟，完善医疗资源融合共享、分级诊疗机制。加快推进市第一医院城东新院二期、福医大附二院东海院区、妇产医院、老年医院等13个卫生重点项目。加强县级公立医院五大核心专科和五大薄弱学科建设，推行家庭医生签约服务。引进培养卫生人才，抓好医风医德建设。做好重大疾病和慢性病防控。争创全国中医药先进市。支持社会办医多元化发展。推动计生服务管理转型，提升妇幼健康服务能力，发挥好计生协会作用。

　　努力让群众享有更优质的养老服务。优先发展居家养老服务，落实新建城区和住宅区配建养老服务设施制度，完善社区养老服务站、日间照料中心；建设150个农村居家养老服务站，提高乡镇养老床位使用率。引导社会力量兴建养老机构，培育专业化服务组织。建设覆盖全市的老年人信息数据库，开展智慧养老试点。深化医养结合，健全医疗卫生、养老机构合作机制，促进医、养、康、护一体化。

　　努力让群众享有更可靠的社会保障。加大对灵活就业、创业项目的扶持力度，推动高校毕业生、退伍军人和城乡就业困难人员，实现更高质量就业创业。深入实施全民参保计划，稳步提高各项社会保险待遇水平。坚持“房子是用来住的、不是用来炒的”，坚定不移推进房地产调控，制定稳控房价中长期规划，建立长效机制，努力实现房地产健康发展、实体经济转型升级、市民住房条件改善“三赢”局面。抓好保障性安居工程，加快石结构房屋、农村危房改造。加强失地失海农民保障。推广泉港“救急难”工作机制，建设社会救助综合施救平台。重视发展慈善事业。全面实施妇女儿童发展纲要。完善留守老人、留守儿童关爱服务体系。

　　努力让群众享有更安全的生活环境。全面深化公安改革，推进“雪亮工程”、智慧天网建设，依法打击违法犯罪活动。认真落实信访工作责任制，加强信访法制化建设。落实食品药品安全地方政府负总责制度，深入创建国家食品安全示范城市。抓好重点领域安全生产工作，完善风险分级管控和隐患排查治理体系。支持工会、共青团、妇联等人民团体发挥更大作用。深入开展民族团结进步创建活动。提升依法管理宗教事务水平。对接落实国防动员和军队改革任务，努力解决好军人后路、后院、后代“三后”问题，完善双拥支前运行机制，巩固发展军政军民团结良好局面。

　　三、加强政府自身建设

　　各位代表！中国特色社会主义进入新时代，我们要深入贯彻习近平新时代中国特色社会主义思想，坚决维护习近平总书记在党中央和全党的核心地位，坚决维护以习近平同志为核心的党中央权威和集中统一领导，始终牢记政府前面的“人民”二字，努力做出得到人民认可、经得起历史检验的工作业绩。

　　转型跨越要有新担当、新作为。进一步解放思想、开拓创新，以功成不必在我的责任担当，以一往无前的奋斗姿态，加快实施赶超三年行动计划，始终保持旺盛的“走前列”势头。突出路线图思维、项目化运作和专业化推进，狠抓重大产业、重大项目、重大改革，强力突破事关全局的重点领域和关键环节。坚持马上就办，拿出真抓的实劲、敢抓的狠劲、善抓的巧劲、常抓的韧劲，确保上级和市委决策部署落地见效。强化正向激励，注重在招商、征迁、企业帮扶、脱贫攻坚、污染治理、风险化解等一线繁重岗位，锤炼一支敢于担当、踏实做事的干部人才队伍。

　　为民惠民要有新行动、新成效。要带着深厚的感情，站在群众的立场，从人民群众关心的事情做起，坚持财政支出优先投向民生事业。高标准实施36件为民办实事项目。深化“放管服”改革，抓好市行政服务中心搬迁工作，进一步推动集中审批和统一监管；持续规范行政审批自由裁量权，深化行政审批标准化改革，推行自然人和法人审批服务事项分类简政放权；实行受理与审批环节分离，推广24小时自助服务，打造百姓“15分钟办事圈”；全面推行“一门一号一窗一网”新服务模式，建设“互联网+政务服务”一体化平台，致力打造一流营商环境。

　　从严治政要有新要求、新形象。推进政府系统全面从严治党，开展“不忘初心、牢记使命”主题教育，深入落实中央八项规定及实施细则精神，坚决纠正“四风”突出问题特别是形式主义、官僚主义新表现。坚持用法治思维和法治方式推进政府工作，扎实推进行政诉讼、行政复议和政务公开工作，深化府院联动互动，加强“七五”普法。依法接受人大法律监督、工作监督，落实重大决策出台前向人大报告制度，主动接受政协民主监督，认真办理人大代表建议和政协提案，充分听取各民主党派、工商联、无党派人士和各人民团体的意见，自觉接受监察和审计监督。落实党风廉政建设责任制，像精心呵护山清水秀的自然生态一样，大力营造风清气正的政治生态。

　　各位代表！泉州人有精神，企业有根，社会有活力。推进泉州新一轮跨越发展，每一份力量都不可或缺，每一天时间都不能耽误。让我们紧密团结在以习近平同志为核心的党中央周围，在中共泉州市委领导下，全社会行动起来，尽锐出战、爱拼敢赢，为加快开创“五个泉州”新局面而努力奋斗！
